# Allometry of carbon and nitrogen content and growth rate in a diverse range of coccolithophores

**DOI:** 10.1093/plankt/fbab038

**Published:** 2021-05-20

**Authors:** Naomi Villiot, Alex J Poulton, Elizabeth T Butcher, Lucie R Daniels, Aimee Coggins

**Affiliations:** The Lyell Centre for Earth and Marine Science and Technology, Heriot-Watt University, Research Avenue South, Edinburgh, EH14 4AS, UK; The Lyell Centre for Earth and Marine Science and Technology, Heriot-Watt University, Research Avenue South, Edinburgh, EH14 4AS, UK; Ocean and Earth Science, National Oceanography Centre Southampton, University of Southampton, Waterfront Campus, Southampton, SO18 3ZH, UK; Ocean and Earth Science, National Oceanography Centre Southampton, University of Southampton, Waterfront Campus, Southampton, SO18 3ZH, UK; Ocean and Earth Science, National Oceanography Centre Southampton, University of Southampton, Waterfront Campus, Southampton, SO18 3ZH, UK; Atmospheric and Ocean Sciences, College of Life and Environmental Sciences, University of Exeter, Prince of Wales Road, Exeter EX4 4PS, UK

**Keywords:** coccolithophores, comparative biochemistry, ecology, elemental stoichiometry

## Abstract

As both photoautotrophs and calcifiers, coccolithophores play important roles in ecosystems and biogeochemical cycles. Though some species form blooms in high-latitude waters, low-latitude communities exhibit high diversity and niche diversification. Despite such diversity, our understanding of the clade relies on knowledge of *Emiliana huxleyi*. To address this, we examine carbon (C) and nitrogen (N) content of strains (*n* = 9) from the main families of the calcifying Haptophyceae, as well as allometry and cell size frequency across extant species. Coccolithophore cell size is constrained, with ~71% of 159 species smaller than 10 μm in diameter. Growth rates scale with cell biovolume (μ = 1.83 × cell volume^−0.19^), with an exponent close to metabolic theory. Organic carbon (C) per cell is lower than for other phytoplankton, providing a coccolithophore-specific relationship between cell organic C content and biovolume (pg C cell^−1^ = 0.30 × cell volume^0.70^). Organic C to N ratios (~8.3 mol:mol) are similar to other phytoplankton, implying little additional *N* cost for calcification and efficient retention and recycling of cell N. Our results support observations that coccolithophores are efficient competitors in low-nutrient conditions, able to photosynthesize, calcify and run the routine metabolic machinery necessary without any additional need for N relative to noncalcifying algae.

## INTRODUCTION

Coccolithophores are marine eukaryotic algae characterized by their unique ability to produce calcium calcite (CaCO_3_) scales, called coccoliths, which are extruded to the outside of the cell during at least one stage of their life cycle (Probert and Houdan, 2004). As photoautotrophs, coccolithophores contribute to pelagic primary production, fixing carbon dioxide (CO_2_) into organic matter, whereas their intracellular calcification also releases CO_2_. Coccolithophores have key roles in the marine carbon cycle, with CaCO_3_ enhancing the flux of carbon (C) to the deep sea via the biological carbon pump (Armstrong *et al*., 2002; Klaas and Archer, 2002) and CaCO_3_ production and export contributing to the carbonate-counter pump (Zeebe and Wolf-Gladrow, 2001). On a cellular basis, coccolithophores may fix as much (or more) inorganic carbon (CaCO_3_) as their cellular inventory of organic carbon (Monteiro *et al*., 2016), although the ratio varies between species (e.g. Daniels *et al*., 2014a). As well as C, coccolithophores are also important in the oceanic uptake and recycling of elements such as nitrogen (N) and phosphorus, which are required for photosynthesis, cell division and metabolism.

Species of coccolithophores share the same basic algal cell structure surrounded by a coccosphere that varies in shape, architecture and crystallography, and the number and arrangement of coccoliths (Monteiro *et al*., 2016). Coccolithophores possess a haplodiplontic life cycle that alternates between haploid and diploid generations (Houdan *et al*., 2004), with the haploid motile cell either naked or possessing a coccosphere of simple CaCO_3_ crystals called holococcoliths (HOT), and the nonmotile diploid cell possessing more complex heterococcoliths (HET) (Cros *et al*., 2000; Geisen *et al*., 2002; Young *et al*., 1999). These two distinct coccolith-producing phases are characterized by different ultrastructures, morphologies and behaviors (Young *et al*., 2003), with the alternation between life-stages widening the ecological niche of coccolithophore species (de Vries *et al*., 2021).

Although the coccolithophore biomineralization process is still not fully understood, we know that in the case of HET species coccoliths are produced (up to ~1 to 2 coccoliths per hour) in intracellular Golgi-derived vacuoles (Monteiro *et al*., 2016). The endomembrane system likely supplies the substrates for calcification, whereas biomolecules (e.g. coccolith-associated polysaccharides (CAPs) and proteins) regulate the process (Taylor *et al*., 2017). Coccolithophore biomineralization requires some of the greatest sustained transcellular ion fluxes reported in eukaryote cells (Brownlee and Taylor, 2004; Brownlee *et al*., 2015), with around 20–30% of the total photosynthetic energy budget associated with calcification (Monteiro *et al*., 2016).

The cellular nutrient use for calcification is not currently clear. Monteiro *et al*. (Monteiro *et al*., 2016) concluded that the nutrient cost of calcification was minimal as the extruded CAPs have very low N and P content, and coccolith production continues after cell division ceases due to nutrient limitation (see Monteiro *et al*., 2016 and references therein). However, this perspective fails to consider any nutrient involvement in structural or functional biomolecules; for example, the protein matrix for CaCO_3_ crystal growth (Mackinder *et al*., 2011), the membrane trafficking system for coccolith extrusion (Lee *et al*., 2015), or nucleoside phosphates and polyphosphates involved in cell metabolism (Merchant and Helmann, 2012). Although calcification may have no clear nutrient “cost”, this is not to say that nutrients are not involved in the metabolism of producing coccoliths and extruding them into the external environment.

Coccolithophores are one of the most comprehensively described groups of oceanic nanoplankton, easily identified by their characteristic cell morphologies, and thus are an ideal group for investigating the role of biodiversity in plankton ecology (Young *et al*., 2003). However, regardless of their high variability in cell shape, calcification rate (Daniels *et al*., 2018; Poulton, 2019) and taxonomic diversity (Young *et al*., 2003), most of our understanding of coccolithophore biology relies on knowledge of a single species, *Emiliana huxleyi* (Aloisi, 2015; Taylor *et al*., 2017).

Cell size and elemental composition are known to influence processes from individuals and populations to ecosystems (Sterner and Elser, 2002). Phytoplankton cell size alters metabolic rates and ecological functions (Chisholm, 1992; Finkel *et al*., 2010; Aloisi, 2015; Garcia *et al*., 2016): including rates of growth, photosynthesis and respiration (Marañón, 2019); the efficiency of resource acquisition, such as light and nutrients (Raven, 1984; Finkel, 2001; Shuter, 1978; Sand-Jensen *et al*., 1995); sinking rates (Smayda, 1970); and mortality factors (Kiørboe *et al*., 1993). Cell size also influences the cellular concentrations of elements such as C and N (Menden-Deuer and Lessard, 2000; Geider and La Roche, 2002), as well as metabolic rates.

Allometric theory predicts that metabolic rates scale with body size (in units of biomass or volume) where individual metabolic rates (R), or cell-specific metabolic rates in the case of microorganisms, scale as R = a^*^W^b^ (e.g. Marañón, 2019). When biomass-specific metabolic rates are considered, the scaling exponent b-1 implies that the pace of metabolism becomes slower in larger cells following a “3/4 rule” (Finkel *et al*., 2004). Therefore, the size-scaling exponent b takes a value of ~−1/4, although there is variability in the exponents associated with phytoplankton (from −0.1 to −0.3; Marañón, 2019). Previous studies on coccolithophores have observed values with a range from −0.11 (Aloisi, 2015) to −0.32 (Buitenhuis *et al*., 2008). As the quantitative relationship between cell size and physiological rates is key to models of phytoplankton productivity and community structure (e.g. Andersen *et al*., 2016; Lindemann *et al*., 2017; O’Brien *et al*., 2016), determining the relationship for the coccolithophore clade is needed to accurately model their growth and responses to environmental conditions.

Phytoplankton cell shape and surface-area-to-volume (SA:V) ratios are morphological traits that directly relate to the fitness of the individual. Together with cell size, they affect growth, metabolism and access to resources (Litchman and Klausmeier, 2008; Naselli-Flores and Barone, 2011). Cell volume relates to nutrient uptake rates of small phytoplankton, whereas surface area becomes important for larger cells (Dao, 2013). Both cell volume and surface area have long been considered the major factors for nutrient uptake rates, transformation and allocation of energy and materials (Okie, 2013). Therefore, to understand the processes that control plankton community structure, as well as those that determine whether allometric mechanisms of multicellular macro-organisms also take place in unicellular microorganisms, we need to further examine the relationship between phytoplankton cell size, SA:V and metabolic rates (DeLong *et al*., 2010).

A number of laboratory studies have examined phytoplankton cellular elemental content, in terms of evolutionary inheritance (Quigg *et al*., 2003, 2011), the biomolecular basis (Geider and La Roche, 2002; Liefer *et al*., 2019), and variability under different environmental conditions and between different phyla (Geider and La Roche, 2002; Garcia *et al*., 2018). However, specific data on coccolithophore cell size and elemental content are constrained to just a few studies and a few taxa (e.g. cell size and metabolic rates, Aloisi, 2015; C:N:P stoichiometry, Quigg *et al*., 2003, 2011; Gerecht *et al*., 2014; Garcia *et al*., 2018), especially when compared to the data published on diatoms, dinoflagellates and microscopic green algae. Comparative analyses of the biology of coccolithophore species, fully representative of their diversity, are essential (Taylor *et al*., 2017) to understand their physiology, ecology and biogeochemical roles within plankton communities, but are currently lacking.

Here we examine the size-scaling of growth rate of coccolithophore cultures, explore whether cellular elemental content (C, N) is conserved within the coccolithophore clade, and compare it with other phytoplankton taxa. Specifically, we examine C to volume relationships (e.g. Menden-Deuer and Lessard, 2000) and cellular C and N content (e.g. Geider and La Roche, 2002). We also perform a meta-analysis of cell size data of ~160 extant coccolithophore species and draw conclusions on coccolithophore cell size distribution in comparison with other phytoplankton groups.

## METHODS

### Coccolithophore cell size distribution meta-analysis

We built a dataset of 159 coccolithophore species representative of 18 taxonomic families based on coccolith and coccosphere measurements, and taxonomic information taken from Young *et al*. (2003), as well as pers. comms. from both J Young and P Bown (University College London). Inner cell diameters were estimated from coccosphere diameter based on coccolith thickness, shape and the number of layers of coccoliths (Young *et al*., 2003).

### Biochemical analysis dataset

Biochemical analyses were obtained from seven coccolithophore species (nine strains) cultivated in the laboratory, and were used to examine coccolithophore allometry and cellular elemental content.

The nine isolates selected from the Roscoff Culture Collection (RCC; France) were chosen to cover a wide range of coccolithophore cell diameters and to be representative of coccolithophore taxonomic diversity. They include *Reticulofenestra parvula* strain RCC 4036; *Emiliania huxleyi* strains RCC 1731 and RCC 1228 (both morphotype A; Poulton, unpublished obs.); *Gephyrocapsa muellerae* strain RCC 3370; *Gephyrocapsa oceanica* strain RCC 1334; *Calcidiscus leptoporus* strains RCC 1130 and RCC 1135; *Syracosphaera pulchra* strain RCC 1461; and *Coccolithus braarudii* strain RCC 1198 (see Table I for the taxonomic nomenclature). This provides coverage of four taxonomic families: Calcidiscaceae, Coccolithaceae, Noelaerhabdaceae and Syracosphaerceae.

**Table I:** Taxonomic nomenclature and sampling location, cell diameters (μm), cell surface-areas (μm^2^), cell volumes (μm^3^) and surface-area-to-volume (SA:V) ratios (μm^−1^) of the nine coccolithophore strains cultivated in this study for biochemical analyses. Values in parenthesis indicate standard deviations

Species	Strain RCC ID	Sampling location	Order	Family	Cell diameter (μm)	Cell surface-area (SA) (μm^2^)	Cell volume (μm^3^)	Surface-area-to-volume (SA:V) ratio (μm^−1^)
*Reticulofenestra parvula*	RCC 4036	South East Pacific	Isochrysidales	Noelaerhabdaceae	3.0 (±0.1)	28.6 (±1.9)	14.4 (±1.4)	2.0 (±0.1)
*Emiliania huxleyi*	RCC 1731	South Pacific	Isochrysidales	Noelaerhabdaceae	4.0 (±0.0)	50.3 (±0.0)	33.5 (±0.0)	1.5 (±0.0)
*Emiliania huxleyi*	RCC 1228	English Channel	Isochrysidales	Noelaerhabdaceae	4.51 (±0.1)	63.9 (±3.0)	48.0 (±3.4)	1.3 (±0.0)
*Gephyrocapsa muellerae*	RCC 3370	Chile Coast	Isochrysidales	Noelaerhabdaceae	5.0 (±0.1)	78.2 (±2.9)	65.0 (±3.7)	1.2 (±0.0)
*Gephyrocapsa oceanica*	RCC 1314	French Coast	Isochrysidales	Noelaerhabdaceae	7.7 (±0.0)	184.0 (±2.0)	234.6 (±3.9)	0.8 (±0.0)
*Calcidiscus leptoporus*	RCC 1130	South Atlantic	Coccolithales	Calcidiscaceae	10.4 (±0.0)	338.8 (±2.5)	586.4 (±6.5)	0.6 (±0.0)
*Syracosphaera pulchra*	RCC 1461	Tyrrhenian Sea	Syracosphaerales	Syracosphaeraceae	11.4 (±0.2)	409.6 (±13.2)	779.6 (±31.4)	0.5 (±0.0)
*Calcidiscus leptoporus*	RCC 1135	South Atlantic	Coccolithales	Calcidiscaceae	13.3 (±0.1)	558.9 (±9.6)	1242.7 (±32.0)	0.5 (±0.0)
*Coccolithus braarudii*	RCC 1198	English Channel	Coccolithales	Coccolithaceae	15.5 (±0.1)	751.2 (±6.00)	1936.1 (±23.2)	0.4 (±0.0)

Cultures of *E. huxleyi* strain RCC 1731 were grown in batch culture from low cell densities in ventilated sterile culture flasks in sterile-filtered K/2 medium, a modified recipe of K medium (Keller *et al*., 2007; Gerecht *et al*., 2014), using aged (dark, 6 months) natural sea water from the Southern Ocean, whereas all other culture duplicates were grown in filtered sterilized K/20 medium, a 10-fold dilution of K/2 medium, with an identical media N:P ratio (i.e. 16:1). All cultures were maintained under optimum growth conditions following recommendations from the RCC (I. Probert, pers. comm.); for example, *C. braarudii* is known to be sensitive to high light. Cultures were grown under a 14:10 light/dark (L/D) cycle at 16°C for *C. braarudii* strain RCC 1198 and 18°C for all the other strains and under replete nutrient and irradiance conditions: 35 μE m^−2^ s^−1^ for *C. braarudii* strain RCC 1198 and average irradiance conditions of 125 μE m^−2^ s^−1^ for all other strains.

Growth of the cultures was monitored everyday by cell counting using either light microscopy with a 1-mL Sedgewick rafter cell (Pyser-SGI, Kent, UK) for the large cells (RCC 1198, RCC 1130, RCC 1135 and RCC 1461) (Langer *et al*., 2006) or a Multisizer™ 3 Coulter Counter ® (Beckman Coulter Ltd, High Wycombe, Buckinghamshire, UK) for the small species (RCC 1228, RCC 1314, RCC 3370 and RCC 4036). For *E. huxleyi* strain RCC 1731 cell counting was performed using a hemocytometer (Neubauer improved, Blaubrand, Germany). Daily sampling ensured the samples were taken during the midexponential phase to avoid potential artifacts of nutrient or carbon limitation on cell size and cellular elemental content (Langer *et al*., 2009; Daniels *et al*., 2014a). Cell densities were plotted against time and growth rates (μ) were calculated by exponential regression (Langer *et al*., 2006; Daniels *et al*., 2014a). To ensure cell division was complete, cell analysis and counting took place 3 hours into the light cycle (Müller *et al*., 2008). Cultures were harvested for cellular elemental composition and the biometric measurements during the midexponential phase for each species, before reaching nutrient-limited conditions.

### Direct measurements of cell size

Cell size of the nine strains examined for in-depth biochemical analyses (see Culturing section) were measured on triplicate samples from midexponential culture material filtered onto cellulose nitrate filters (22 mm diameter, 0.8 μm pore size) and oven dried overnight at 50°C. Permanent slides were prepared by mounting filters onto glass slides using low viscosity optical adhesive (No. 74, Norland Products, Cranberry, New Jersey, USA) (Poulton *et al*., 2010). One hundred cells per slide were measured to obtain an average cell diameter for each species using an eyepiece graticule calibrated at 0.1 mm, 0.01 mm and 0.05 mm under an Olympus BX53F polarizing light microscope (×100, oil immersion). The surface area (A) and volume (V) of each species were then calculated using the average diameter (d) of the replicates, with all species determined to have a spherical cellular shape (i.e. A = π ^×^ d^2^, Hillebrand *et al*., 1999; V = (π/6) ^×^ d^3^, Sun and Liu, 2003). Throughout the article when the terms “cell size”, “cell diameter”, “cell surface-area-to-volume ratio” and “cell volume” are used, we refer to the inner (organic) cell of coccolithophores which does not include the coccoliths.

### Cellular elemental content

Triplicate samples for the analysis of particulate organic carbon (POC), total particulate carbon (TPC) and particulate organic nitrogen (PN) were collected under low pressure vacuum filtration (100 mm Hg) onto precombusted (450°C, 12 hours) Whatman GF/F filters (25 mm diameter, 0.7 μm effective pore size). For POC/PN, filters were rinsed with 1% hydrochloric acid (HCl) after filtration to remove particulate inorganic carbon (PIC). Filters for TPC and POC/PN were oven dried at 50°C overnight and then folded into tin capsules, pelleted and placed into a 96-well microplate. The microplate was stored in a desiccator until analysis with a Thermo Flash 2000 Carbon-Hydrogen-Nitrogen-Sulphur (CHNS) analyzer (Thermo Fisher Scientific Inc., Waltham, MA, USA). PIC was determined from the difference between TPC and POC of the cultures.

### Data analysis

Data were analyzed in RStudio (version 1.2.1335) (RStudio, Boston, MA, USA) using Envstats to perform basic univariate statistical analyses. Figures were prepared using Origin Pro (OriginLab Corporation, Northampton, MA, USA). Our biochemical data on coccolithophores were compared with published data on species of diatoms (*n* = 18), cyanobacteria (*n* = 4), dinoflagellates (*n* = 8), green algae (*n* = 15), red algae (*n* = 4) and coccolithophores (*n* = 5) by plotting C:N ratios (see Fig. 5).

## RESULTS

### Coccolithophore size spectra and allometry

**Fig. 1:**
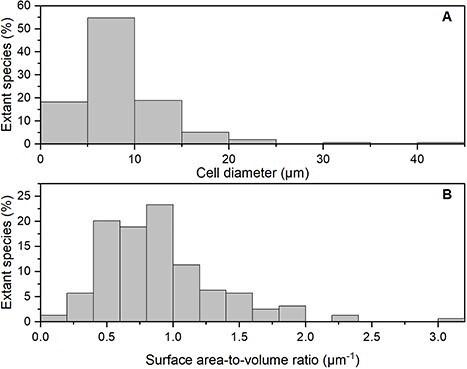
Frequency histogram of the percentage of extant species (based on Young *et al*., 2003) in different bins of (A) cell size (μm) and (B) surface area to volume ratio (μm^−1^).

**Fig. 2:**
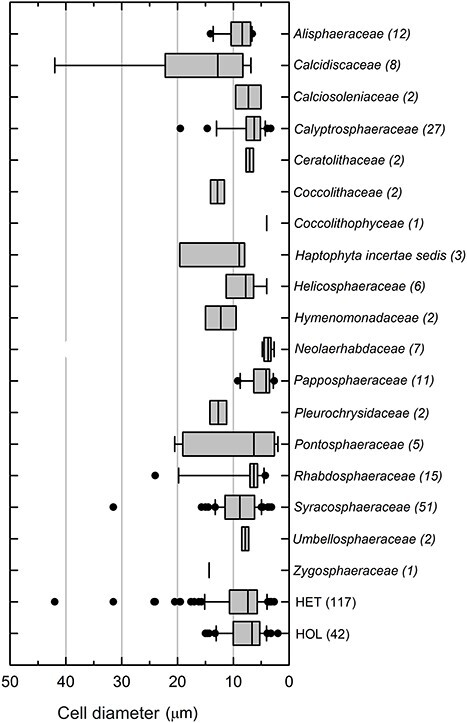
Boxplots of cell size distribution for different coccolithophore families and coccolith types (Heterococcoliths, HET; Holococcoliths, HOL). Values in parentheses represent the number of species in each family or type. Boxes cover the upper and lower quartiles (25–75th percentiles), whiskers are the standard deviations and outliers are indicated as black points.

The 159 coccolithophore species examined in the meta-analysis have cell diameters (μm) ranging from 2 to 42 μm (Fig. 1A). The smallest species was *Sphaerocalyptra quadridentata* (Pontosphaeraceae), whereas the largest species was *Hayaster perplexus* (Calcidiscaceae). The majority of coccolithophore species (~71%) are smaller than 10 μm in diameter, whereas approximately half of the species (~52%) have cell dimensions between 5 and 10 μm (Fig. 1A). The geometric mean of cell diameters is 7.6 μm (geometric SD ± 1.7 μm). Examination of the cell size distribution between different coccolithophore families (Fig. 2) shows that in general most families have median cell sizes less than 10 μm, though there are examples of species larger than 10 μm in many families. However, species within some families have limited cell size distributions; for example, the family in which the common species *Emiliania huxleyi* is found (Neolaerhabdaceae) has a very limited cell size distribution around 4 μm, which is smaller than many other coccolithophore families (Fig. 2). Dividing the coccolithophores between diploid (possessing HET) and haploid (possessing HOL) life stages shows no appreciable difference in cell size, with both stages having similar median cell sizes around 7–8 μm (Fig. 2).

Based on the geometric mean coccolithophore cell diameter of the meta-analysis dataset (7.6 μm), small-celled species (i.e. diameters <7.6 μm) from the biochemical analysis dataset are *Reticulofenestra parvula* (strain RCC 4036), *Emiliania huxleyi* (both strains RCC 1228 and RCC 1731), *Gephyrocapsa muellerae* (strain RCC 3370) and *Gephyrocapsa oceanica* (strain RCC 1314). Large celled species (i.e. cell diameter >7.6 μm) included in the biochemical analysis are *Calcidiscus leptoporus* (both strains RCC 1130 and RCC 1135), *Syracosphaera pulchra* (strain RCC 1461) and *Coccolithus braarudii* (strain RCC 1198). Average cell volumes and standard deviations for the nine strains examined are included in Table I. The cell diameters for the strains included in the biochemical analysis dataset ranged from 3.0 (*R. parvula*) to 15.5 μm (*C. braarudii*).

With SA:V ratios being informative in terms of nutrient uptake for plankton, we also calculated SA:V ratios for the 159 species for which we have cell diameters, assuming the majority of cells are spherical. Ratios of SA:V for coccolithophores range from 0.14 to 3.00 (Fig. 1B), with ~72% of the values above 0.6 μm^−1^ and a geometric mean ratio of 0.79 μm^−1^ for the whole clade. As expected, the smallest species corresponded to the largest SA:V ratio and the largest species corresponded to the smallest SA:V ratio (*S. quadridentata* and *H. perplexus*, respectively).

**Fig. 3:**
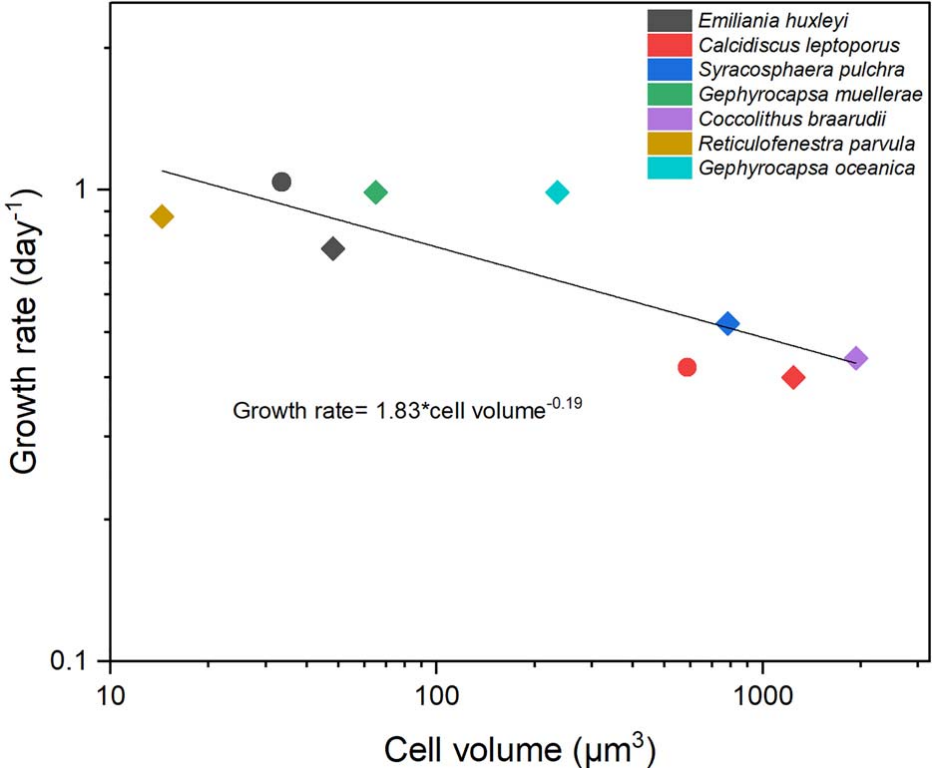
Log–log relationship between growth rates (d^−1^) and cell volumes (μm^3^) for the nine coccolithophore strains cultivated in this study for biochemical analyses. Colors differentiate species whereas symbols differentiate strains of the same species. Solid line is a linear regression (μ = 1.83 × cell volume^−0.19^, *r^2^* = 0.70, *P* < 0.05, *n* = 9) for all species.

For the nine strains cultured in laboratory conditions and examined in terms of cellular elemental composition, SA:V ratios spanned from 0.34 to 1.99 (see Table I). Volumetrically, these species ranged from 14.4 μm^3^ to 1936.1 μm^3^, with a ~130-fold difference in cell volume (Table I). Cell sizes for the culture strains were in agreement with those for the same species in the cell size meta-analysis, showing little if any shrinkage related to the drying and mounting of cells. Specific growth rates of the nine cultured coccolithophores are plotted against cell volume in Fig. 3, with growth rates ranging from 0.40 d^−1^ (*C.leptoporus* strain RCC 1135) to 1.04 d^−1^ (*E. huxleyi* strain RCC 1731). Growth rates (log, d^−1^) negatively scale with increasing cell volume (log, μm^3^) with the relationship for all species in Fig. 3 having a power exponent of −0.19 (SE ± 0.05) (μ = 1.83 × cell volume^−0.19^; *r*^2^ = 0.70; *P* < 0.05). Comparing our allometric exponent (−0.19) with previously reported exponents of coccolithophore allometry (−0.11, Aloisi, 2015; −0.32, Buitenhuis *et al*., 2008) and phytoplankton allometry (−0.09, Marañón *et al.*, 2013), as well as metabolic theory (−0.25), using a Kruskal–Wallis ANOVA for nonparametric data revealed statistically significant differences (*P* < 0.05).

### Coccolithophore cellular elemental content

**Fig. 4:**
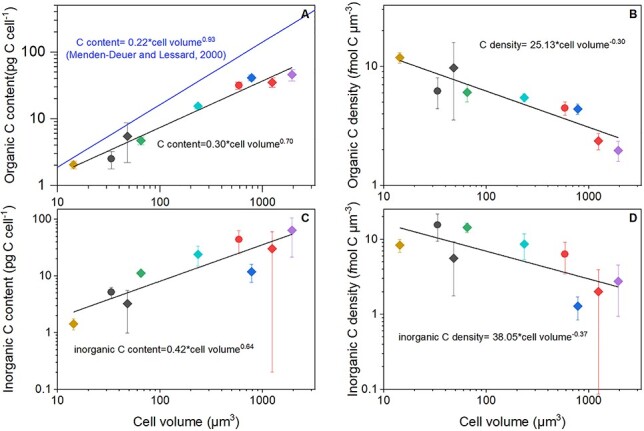
Log–log relationships for cell volume, cellular (A) organic carbon content (pg C cell^−1^) and (B) organic carbon density (*f*mol C μm^−3^) for the nine coccolithophore strains cultivated in this study for biochemical analyses. Colors differentiate species whereas symbols differentiate strains of the same species. Vertical error bars are standard deviations. Solid black lines indicates linear regressions for all species: (A) cell organic C content = 0.30 × cell volume^0.70^ (*r^2^* = 0.97, *P* < 0.05, *n* = 9); (B) cell organic C density = 25.13 × cell volume^−0.30^ (*r^2^* = 0.84, *P* < 0.05, *n* = 9); (C) cell inorganic C content = 0.42 × cell volume^0.64^ (*r^2^* = 0.81, *P* < 0.05, *n* = 9); and (D) cell inorganic C density = 38.05 × cell volume^−0.37^ (*r^2^* = 0.57, *P* < 0.05, *n* = 9). Blue line on panel (A) indicates the relationship from Menden-Deuer and Lessard (Menden-Deuer and Lessard, 2000).

Elemental content per cell for the nine coccolithophore strains cultured are given in Table II. Cell organic nitrogen (N) content exhibited a ~30-fold difference between the species, from 0.02 (SD; ± 0.001) pmol C cell^−1^ for *R. parvula* to 0.62 (±0.06) pmol C cell^−1^for *S. pulchra*. Organic carbon (C) content ranged with a ~20-fold difference across this cell size range, from 0.17 (SD ± 0.02) to 3.82 (±0.74) pmol C cell^−1^. This range in cell organic C content corresponded to the smallest (*R. parvula*) and largest (*C. braarudii*) species considered, respectively. Organic C content (log, pg C cell^−1^) positively scaled with cell volume (log, μm^3^) (Fig. 4A) with a power exponent of 0.70 (SE ± 0.05) (cell organic C content = 0.30 × cell volume^0.70^; *r*^2^ = 0.97; *P* < 0.05). Numerous relationships between organic C content and cell volume exist in the literature (Table IV) and a comparison with these published relationships highlights that our relationship implies that coccolithophores tend to have lower cell organic C content per unit cell volume than other phytoplankton groups. Our log–log relationship of coccolithophore cellular organic C content versus cell volume (*n* = 9) and published C to biovolume relationships (Table IV) were statistically different (Kruskal–Wallis ANOVA; *P* < 0.05). In our study, cellular organic C densities (*f*mol C μm^−3^) range from 1.97 (SD ± 0.38) to 11.84 (±1.18) for the strains examined in our culture experiments (Fig. 4B), with a log–log relationship between organic C density and cell volume showing a negative slope with an exponent of −0.30 (SE ± 0.05) (cell organic C density = 25.13 × cell volume^−0.30^; *r*^2^ = 0.84; *P* < 0.05).

**Table II:** Cellular elemental composition (C and N) of the nine coccolithophore strains cultivated in this study for biochemical analyses. Cell content is reported in pmol cell^−1^ for particulate organic carbon (POC), particulate inorganic carbon (PIC) and particulate organic nitrogen (PON)

Species and strain RCC ID	POC content		PIC content		PON content	
	pmol_POC_ cell^−1^	SD	pmol_PIC_ cell^−1^	SD	pmol_PON_ cell^−1^	SD
*R. parvula* (RCC 4036)	0.17	0.02	0.12	0.03	0.02	0.00
*E. huxleyi* (RCC 1731)	0.21	0.06	0.52	0.21	0.05	0.01
*E. huxleyi* (RCC 1228)	0.45	0.27	0.27	0.19	0.06	0.03
*G. muellerae* (RCC 3370)	0.39	0.05	0.93	0.12	0.05	0.01
*G. oceanica* (RCC 1314)	1.29	0.10	2.03	0.78	0.19	0.02
*C. leptoporus* (RCC 1130)	2.62	0.33	3.69	1.62	0.35	0.03
*S. pulchra* (RCC 1461)	3.43	0.33	1.00	0.35	0.62	0.06
*C. leptoporus* (RCC 1135)	2.94	0.47	2.55	2.54	0.39	0.14
*C. braarudii* (RCC 1198)	3.82	0.74	5.35	3.55	0.26	0.11

The cellular inorganic C content of the nine strains examined ranged from 0.12 (SD ± 0.03) to 5.35 (±3.55) pmol C cell^−1^ (Table II), this range in cell content corresponded to the smallest (*R. parvula*) and largest (*C. braarudii*) coccolithophore species examined, with a ~40-fold difference in inorganic C content across this cell size range. Inorganic C content (log, pg C cell^−1^) positively scaled with cell volume (log, μm^3^) in the species examined in our study (Fig. 4C), with a power exponent of 0.64 (SE ± 0.12) (cell inorganic C content = 0.42 × cell volume^0.64^; *r*^2^ = 0.81; *P* < 0.05). Cellular inorganic C densities (*f*mol C μm^−3^) range from 1.28 (SD ± 0.38) to 15.58 (±6.20) (Fig. 4D) and corresponded to *S. pulchra* and *E. huxleyi* strain RCC 1731, with a log–log relationship between inorganic C density and cell volume showing a negative slope with an exponent of −0.37 (SE ± 0.12) (C density = 38.05 × cell volume^−0.37^; *r*^2^ = 0.57; *P* < 0.05).

### Coccolithophore elemental molar ratios

**Fig. 5:**
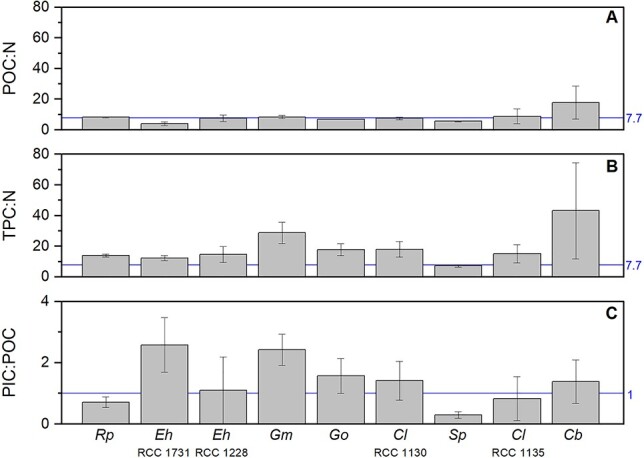
Average elemental stoichiometric ratios (mol mol^−1^) for the nine coccolithophore strains cultivated in this study. Ratios are given for: (A) particulate organic carbon (POC) to nitrogen (N); (B) total particulate carbon (TPC) to nitrogen (N); (C) particulate inorganic carbon (PIC) to particulate organic carbon (POC). Vertical error bars are standard deviations (*n* = 3). Blue horizontal lines indicate the average values for the carbon to nitrogen (C:N) from Geider and La Roche (Geider and La Roche, 2002). *Reticulofenestra parvula* (Rp); *Emiliania huxleyi* (Eh); *Gephyrocapsa muellerae* (Gm); *Gephyrocapsa oceanica* (Go); *Calcidiscus leptoporus* (Cl); *Syracosphaera pulchra* (Sp); and *Coccolithus braarudii* (Cb).

Species-specific molar ratios of the cellular C and N constituents with respect to both organic C and total C (i.e. organic + inorganic) are represented in Fig. 5 and values are given in Table III. Average cellular ratios (mol:mol) of organic C to organic N ranged from 4.01 (SD ± 1.22) to 17.82 (±10.80), corresponding to *E. huxleyi* (RCC 1731) and *C. braarudii* (RCC 1198), with a geometric mean ratio of 7.67 (±1.49) and an arithmetic average of 8.29 (±3.88) (Fig. 5A; Table III). Average cellular ratios of inorganic to organic C ranged from 0.29 (SD ± 0.11) to 2.58 (±0.89), corresponding to *S. pulchra* (RCC 1461) and *E. huxleyi* (RCC 1731), with a geometric mean ratio of 0.98 (SD ± 2.45) and an arithmetic mean of 1.34 (±0.93) (Fig. 5C; Table III).

**Table III:** Elemental ratios (mol mol^−1^) of the nine coccolithophore strains cultivated in this study for biochemical analyses. Average ratios are given to particulate organic carbon (POC) only (POC:Nitrogen (N)) and to total particulate cell carbon (TPC) (TPC:N), which includes both POC and particulate inorganic carbon (PIC)

Species and strain RCC ID						
	POC:N	SD	TPC:N	SD	PIC:POC	SD
*R. parvula* (RCC 4036)	8.13	0.46	13.87	0.92	0.71	0.17
*E. huxleyi* (RCC 1731)	4.01	1.22	12.18	1.68	2.58	0.89
*E. huxleyi* (RCC 1228)	7.60	2.18	14.57	5.23	1.10	1.08
*G. muellerae* (RCC 3370)	8.31	1.09	28.66	6.97	2.43	0.51
*G. oceanica* (RCC 1314)	6.91	0.13	17.74	3.98	1.57	0.57
*C. leptoporus* (RCC 1130)	7.44	0.77	17.99	5.17	1.41	0.64
*S. pulchra* (RCC 1461)	5.54	0.27	7.17	0.82	0.29	0.11
*C. leptoporus* (RCC 1135)	8.83	4.74	15.01	5.93	0.83	0.71
*C. braarudii* (RCC 1198)	17.82	10.80	43.17	31.35	1.38	0.71

Including inorganic C in the total cell C pool gives cellular ratios of total C to organic N ranging from 7.17 (SD ± 0.82) to 43.17 (±31.30), corresponding to *S. pulchra* (RCC 1461) and *C. braarudii* (RCC 1198), with a geometric mean ratio of 18.93 (±10.77) and an arithmetic average of 16.76 (±1.67) (Fig. 5B; Table III). Organic C to N ratios (i.e. ratios calculated from organic C only), even though relatively variable among species, showed no consistent pattern with cell size (not shown). Variability increased when examining the total C to N ratios, but no overall pattern of the ratio varying with cell size was discernible (not shown).

## DISCUSSION

### A constrained coccolithophore cell size spectrum has physiological and ecological implications

Analysis of the cell size dimensions of extant coccolithophore species from the modern ocean show that they have a rather constrained cell size distribution (Figs. 1A and2), with ~71% of extant species having cell diameters smaller than 10 μm and different life stages (HET, HOL) having very similar cell sizes. Phytoplankton cell size groupings range from less than 2 μm for picoplankton, 2–20 μm for nanoplankton, to up to 20–200 μm for microplankton, with key phytoplankton groups forming distinguishable patterns of size distribution (Beardall *et al*., 2009; Finkel *et al*., 2010). Our results highlight that the majority of coccolithophore species are found in the nanoplankton size range. As a group, prymnesiophytes, which includes coccolithophores, are also limited to the nanoplankton (Sommer *et al*., 2017). This is consistent with our observation that coccolithophores are a size-restricted group, with ~52% of the cell diameters from our dataset between 5 and 10 μm. Measurements of fossil coccolithophore species support this modern observation and show that it was true across time intervals and latitude, with a shift toward smaller cells after the Paleocene–Eocene Thermal Maximum (Gibbs *et al*., 2018). In fact, the *Neolaerhabdaceae*, which contains ubiquitous species which are often numerically dominant in the present-day ocean (e.g. *E. huxleyi*, *G. oceanica*), has generally smaller and more restricted cell sizes compared with other coccolithophore families (Fig. 2). Shifts in cell sizes, and associated biomass, likely influence changes in grazing losses, remineralization and ballasting: small cells are more easily recycled in surface waters and less effective ballasting agents (Bach *et al*., 2016; Gibbs *et al*., 2018; Tréguer *et al*., 2018).

Ecological factors and cellular features constrain the minimum cell size of unicellular algae (Raven, 1986). In return, cell size determines the ratio and composition of macromolecules with respect to the cellular concentrations of elements (Geider and La Roche, 2002). Hence, the size-scaling of phytoplankton physiological traits drives the structure and dynamics of phytoplankton communities, as well as their physiology, ecology and evolution (Thompson, 1942; Zeuthen, 1953; Lewis, 1976; Kooijman, 2009). For instance, cell size constrains light acquisition and nutrient uptake in microalgae (Raven, 1984; Chisholm, 1992; Kiørboe *et al*., 1993). Coccolithophores have restricted cell sizes, with ~72% of species having SA:V ratios above 0.6 μm^−1^ (Fig. 1B). Restricted cell sizes likely make coccolithophores good competitors for resource acquisition in low nutrient and low-light environments, with small cells and cell SA:V ratios adapted for passive nutrient diffusion and for limiting the package effect for light harvesting (e.g. Raven, 1984; Chisholm, 1992). In fact, coccolithophores consistently exhibit their highest species diversity in open-ocean oligotrophic environments, such as the central subtropical gyres (e.g. O’Brien *et al*., 2013; Poulton, 2019).

### Coccolithophore size-scaling of growth rate matches basic metabolic theory

When investigating phytoplankton dynamics, the study of the relationship between cell size and metabolic rate is of interest for ecological models (e.g. Finkel *et al*., 2010; Dutkiewicz *et al*., 2020). Our exponent (−0.19) of coccolithophore-specific growth rates versus cell volumes is not as near-isometric as previously reported by Aloisi (−0.11; Aloisi, 2015) or Marañon *et al*. (−0.09; Marañón *et al*., 2013), who both found less size-dependance of variability in growth rates. A study by Lopez-Sandoval *et al*., (2014) demonstrated that phytoplankton metabolism from five phyla (22 species; including only three coccolithophore species) covering seven orders of magnitude in cell size, with many species in the <5 μm cell size range differing significantly from the ¾ rule (Lopez-Sandoval *et al*., 2014). Embedding our new allometric exponent for coccolithophores in models would lead to slightly more of a reduction in growth rates for larger cells relative to smaller cells than for other exponents (Aloisi, 2015; Marañón *et al*., 2013). In fact, a comparative study of growth rates of *E. huxleyi* and the larger *Coccolithus* species under identical temperature and light conditions highlighted a 10–30% difference in growth rates (Daniels *et al*., 2014a).

Phytoplankton allometric relationships can indeed be complex when considering variations in scaling factor among clades or between key functional phytoplankton groups (Beardall *et al*., 2009; Dutkiewicz *et al*., 2020). Measurements of phytoplankton cultures and natural communities report variable exponent values, which are all statistically different from metabolic theory (−0.25), often ranging between −0.1 and −0.3 (Marañón, 2019): for example, −0.13 for diatoms grown at optimal growth temperatures (species *n* = 67, Sarthou *et al*., 2005); −0.15 for all types of algae (*n* = 69, Tang, 1995); and − 0.32 for a smaller set of coccolithophore species (*n* = 5, Buitenhuis *et al*., 2008). Conflicting ways of calculating and expressing growth rates (e.g. maximum growth rates, specific growth rates, or relative growth rates) in the literature, between scientific fields (Sterner and Elser, 2002), and the lack of standards for growth conditions or data collection methods (Marañón, 2015) make it difficult to directly compare published datasets. Our exponent (−0.19) sits between the two coccolithophore-specific allometry relationships already reported (−0.11, Aloisi, 2015; −0.32, Buitenhuis *et al*., 2008), and all three differed significantly from the −1/4 (−0.25) metabolic theory value (Kruskal–Wallis ANOVA; *P* < 0.05), although ours is much closer to this value. Our relationship is based on exponential growth rates (from exponential regression), with replete nutrients and light, across a wider range of coccolithophore species (*n* = 8), including examples from all the major families, than has been examined before, and thus we consider that our exponent (−0.19) better represents the coccolithophores as a clade than previous studies.

Differences in growth rate scaling patterns among phytoplankton groups improves our understanding of competition between groups, including the dominance of certain groups under specific environmental conditions (Dutkiewicz *et al*., 2020). Recent studies have revealed that the highest biomass-specific growth rates (μ_max_) in phytoplankton are achieved by species of intermediate cell size (Finkel *et al*., 2010; Marañón *et al.*, 2013) with a peak around cell volumes of ~100 μm^3^ (Marañón *et al*., 2013). Many bloom-forming phytoplankton species have cell sizes near this peak (Sommer *et al*., 2017), with bloom-forming coccolithophore species such as *Gephyrocapsa oceanica* (~300 μm^3^) (see Rhodes *et al*., 1995), *Emiliana huxleyi* (~50 μm^3^) and *Syracosphaera bannockii* (~400 μm^3^) (see Daniels *et al*., 2014b) all having cell volumes in this range (50–400 μm^3^). Refining exponents of size-scaled growth under both “optimal and natural” oceanic conditions are essential to help improve model predictions and we recommend this as a way forward for the coccolithophore clade.

### Coccolithophores are less (organic) carbon dense than other phytoplankton groups

Our measurements reveal that coccolithophores tend to be less carbon (C) rich than suggested by previous estimations of phytoplankton biomass (e.g. Menden-Deuer and Lessard, 2000; see Fig. 4A and Table IV). As 98% of the variability in C production rates is explained by cell size (Marañón, 2008), the Menden-Deuer and Lessard’s (Menden-Deuer and Lessard, 2000) empirical relationship between C content and cell volume is the basis of many current models and studies of phytoplankton for reconstructing species and community biomass in the modern ocean (e.g. O’Brien *et al*., 2013; Ignatiades, 2017), but also for paleontological studies (e.g. Gibbs *et al*., 2018). Although the study by Menden-Deuer and Lessard (Menden-Deuer and Lessard, 2000) discriminates between diatoms and dinoflagellates, all other types of protists are considered as one group (Table IV), without any distinction for the coccolithophores. Although these authors included a relationship for prymnesiophytes, with a total number of species of 14, the data included only four coccolithophore species.

**Table IV:** Comparison of the C-biovolume relationships (*y = ax^b^*) from several sources where *y* is C (pg cell^-l^); *x* is cell volume (μm^3^); and *a* and *b* (slope) are constants. ^a^ Data collected from Moal *et al*. (1987); Montages *et al*. (1994); Mullin *et al*. (1966); and Verity *et al.* (1992)

References	Equations *y = ax^b^*	Cell volume range (μm^3^)	Total species *n*	Coccolithophore species *n* (strains *n*)	*y* for *x* equals 5 μm
Marañón *et al*. (2013)	pg cell^−1^ = 0.20 × cell volume^0.88^	0.1–2 500 000	22	3 (3)	7.9
Menden-Deuer and Lessard (2000) *for protists excluding diatoms*	pg cell^−1^ = 0.22 × cell volume^0.94^	1.0–1 200 000	91	5 (8)	11.0
Menden-Deuer and Lessard (2000) *for prymnesiophytes^a^*	pg cell^−1^ = 0.23 × cell volume^0.90^	14.2–1 610	14	4 (6)	9.9
Moal *et al*. (1987)	pg cell^−1^ = 0.40 × cell volume^0.83^	31.0–3 328 525	11	0 (0)	12.8
Montages *et al*. (1994)	pg cell^−1^ = 0.11 × cell volume^0.99^	1.0–34 663	30	2 (2)	6.9
Mullin *et al*. (1966)	pg cell^−1^ = 0.51 × cell volume^0.76^	14.2–6 200 000	14	2 (2)	12.3
Verity et al. (1992)	pg cell^−1^ = 0.43 × cell volume^0.86^	1.3–1 407	13	2 (2)	16.0
This study	pg cell^−1^ = 0.30 × cell volume^0.70^	14.4–1936	9	7 (9)	5.6

In general, our coccolithophore-specific relationship predicts lower organic C content per cell than other published relationships (Table IV). For example, taking a cell with a diameter of 5 μm and applying our relationship predicts a cell organic C content value of 5.6 pg C cell^−1^ (Table IV). This value for cellular organic carbon content is close to that measured for coccolithophores (~4.5–10 pg C cell^−1^) in this cell size range (4.4–5.8 μm), with high growth rates (>0.5 d^−1^), by multiple authors (e.g. Arnold *et al*., 2013; Bach *et al*., 2011; Iglesias-Rodriguez *et al*., 2008; Marañón *et al*., 2013; Riegman *et al*., 2000; van Rijssel and Gieskes, 2002). In fact, several of the previously published relationships provide agreeable cell C content for ~5 μm cells (Table IV), and it is only in the higher cell size range that significant differences are likely.

### Calcification and nutrient use

**Fig. 6:**
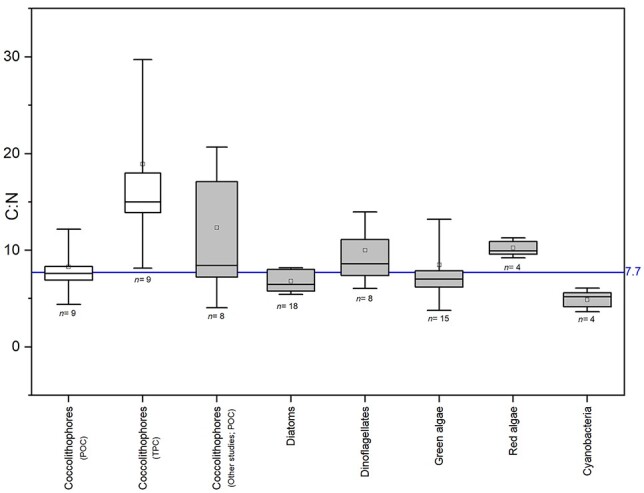
Comparative analysis of carbon to nitrogen (C:N) ratios (mol mol^−1^) for different phytoplankton groups. Coccolithophores (both from this study and from published data); Diatoms; Dinoflagellates; Green algae; Red algae; and Cyanobacteria. Coccolithophore data are from this study for particulate organic carbon (POC) and total particulate carbon (TPC) (white boxes) and from previously published data of POC (gray box; i.e. Garcia *et al*., 2018; Gerecht *et al*., 2014; Liefer *et al*., 2019; Quigg *et al*., 2003, 2011). Box and whisker plots indicate 25 and 75% quantiles with median (solid black lines) and mean (small squares), vertical error bars are standard deviations of the measurements, and the number of observations per group (*n*) is indicated at the top of each box. Solid blue horizontal lines indicate average values from Geider and La Roche (Geider and La Roche, 2002).

Despite variability in cellular elemental content within the group (Figs. 5 and 6), coccolithophores have similar organic C:N ratios as diatoms, dinoflagellates and green algae, lower than red algae and higher than cyanobacteria (Fig. 6). The range (4.01–17.82) and average C:N ratio (8.29 ± 3.88) are well within the ranges described in other phytoplankton studies (see Fig. 6); for example, 6.60 for Redfield (Redfield, 1960), 7.7 for Geider and La Roche (Geider and La Roche, 2002), 7.75 for Ho *et al*. (Ho *et al*., 2003), and 4.85–9.44 for Garcia *et al*. (Garcia *et al*., 2018).

However, coccolithophores unlike other key functional phytoplankton groups, have two pools of cellular C, both organic and inorganic. These two pools of carbon are intrinsic to coccolithophore cells, though they may vary during different life-cycle stages. Our PIC:POC ratios were generally close to 1 (Fig. 5C; Table III), highlighting that coccolithophores fix at least an equal amount of their cellular organic C as CaCO_3_ (Monteiro *et al*., 2016). Hence, the cellular inorganic C pool represent a significant cellular pool of C, which can be equal to (or greater than) the organic C pool. Almost equimolar inventories of inorganic and organic C has important implications for understanding coccolithophore C-metabolism. For instance, Bolton and Stoll (2013) suggested intracellular competition for C from bicarbonate (HCO_3_^−^) fluxes between the site of photosynthesis (the chloroplast) and the site of calcification (the coccolith vesicle). Such competition for C fixation is not found in noncalcifying algae.

With such a significant cellular investment in CaCO_3_, it could be expected that this requires additional metabolic or nutritional resources over and above that found in noncalcifying phytoplankton. However, the organic C to N content of coccolithophores is the same as for other phytoplankton groups, a point made even more obvious by the rough doubling of C:N ratios with the inclusion of their inorganic C content into total cell C (Fig. 6). Not only does this support the notion that calcification requires no additional nutrient cost (senso Monteiro *et al*., 2016), but it highlights that coccolithophores are doing a lot more with this cell N than noncalcifying algae; it seems unreasonable to assume that N-containing biomolecules (e.g. proteins, enzymes) are completely absent from the metabolic processes involved in calcification. Moreover, cell N in coccolithophores must be efficiently retained and internally recycled to allow for its use in both standard metabolic processes and cell maintenance (e.g. resource acquisition, vesicle formation), but also in the processes associated with calcification.

Cell organic C:N ratios similar to other phytoplankton also implies that the N demand for calcification is low and coccolithophores invest most of their cellular N into resource acquisition (e.g. light-harvesting pigments) and growth machinery, rather than in calcification. In fact, previous research has demonstrated that coccolithophores have low half-saturation constants for nitrate uptake and less cell volume dependence on N than other phytoplankton, while still being able to maintain high maximum growth rates (Litchman *et al*., 2007) and coccolith production. In nature, the efficient use of N for cellular processes will also relate to the ecology and biogeography of coccolithophores.

Previous hypotheses regarding bloom formation of coccolithophores by Tyrell and Merico (Tyrell and Merico, 2004) suggested that *E. huxleyi* blooms tend to occur at low P concentrations relative to N (see also: Riegman *et al*., 1992; Aksnes *et al*., 1994; Egge and Heimdal, 1994). Conversely, a later review by Lessard *et al.* (Lessard *et al*., 2005) found that *E. huxleyi* blooms also occur in environments under conditions of N limitation. Blooms may also occur when both N and P are replete and silicic acid limits diatom growth (e.g. Poulton *et al.*, 2013). Hence, multiple factors relate to coccolithophore bloom formation rather than just nutrient availability (Balch, 2018). These studies agree with our data in that they support competitive acquisition and efficient use of N for coccolithophore growth and calcification. Further, a possible solution to observations of coccolithophore blooms and high diversity in N and P limited waters, likely lies within the group’s ability to effectively compete for and utilize organic nutrient sources (Tyrell and Merico, 2004; Poulton *et al*., 2017; Godrijan *et al*., 2020). Benner and Passow (2010) showed that coccolithophores effectively utilize different organic sources of N, while Cermeño *et al*. (Cermeño *et al*., 2011) demonstrated the ability of *C. brarudii* to outcompete a similar-sized diatom under nitrate-limited conditions. Taken together, these different lines of evidence, including our C and N data, highlight the efficient strategies for nutrient capture and retention possessed by coccolithophores.

On a simple C-mass basis, coccolithophores physical fix up to twice the amount of C per unit N into organic and inorganic carbon compared with other phytoplankton groups (Fig. 6). However, while the cellular processes underpinning this C-fixation (photosynthesis, calcification) are an obligate requirement for coccolithophore cell division and growth, the two processes affect seawater carbonate chemistry differently, as well as the carbonate-counter pump and the biological carbon pump (Zeebe and Wolf-Gladrow, 2001). The balance of these different processes occurring in coccolithophore blooms can lead to them acting as CO_2_-sources or CO_2_-sinks, with cellular and community ratios of inorganic and organic production strongly influencing the net effects of calcification and photosynthesis (Robertson *et al*., 1994; Buitenhuis *et al*., 2001; Poulton *et al.*, 2007). Further, effective ballasting of POC export and sequestration in the deep sea by inorganic C (Klaas and Archer, 2002), combined with their use of N to fix both organic and inorganic C, indicates that coccolithophores are important vectors for C fixation, N cycling and export relative to blooms of nonbiomineralized phytoplankton (Bach *et al*., 2016).

## CONCLUSIONS

Our results in terms of coccolithophore cell size, growth rate, carbon and nitrogen content have important implications for their ecology and biogeochemistry:

(1) Coccolithophores are more size-restricted than other phytoplankton groups (e.g. diatoms), with ~71% of 159 extant species smaller than 10 μm in diameter and with most SA:V ratios above 0.6 μm^−1^. This pattern in cell size spectrum potentially gives advantages to coccolithophores in low nutrient and low-light environments when competing with other phytoplankton.

(2) Our coccolithophore size-scaling of growth rate matches quite well with metabolic theory, which differs to several previous studies of phytoplankton allometry, and is likely due to limiting our sampling to coccolithophores and including a wider range of species and families.

(3) A new relationship for the scaling of organic carbon (C) content with cell size specific to coccolithophores is presented and indicates that coccolithophore cells are less organic C dense than other phytoplankton. We also observe a relationship between cell size and cell inorganic C indicating that, for the species we examined, larger cells have higher CaCO_3_ inventories.

(4) Coccolithophore organic carbon to nitrogen (C:N) ratios are generally conserved across different species and provide clear evidence that coccolithophores efficiently use cell N for both standard cell metabolism and resource acquisition as well as CaCO_3_ production. The obligate requirement for CaCO_3_ production for growth requires that coccolithophores have developed efficient strategies for N acquisition and retention as they are successful competitors in low-nutrient waters at both low- and high-latitudes.
